# Age and antiplatelet de-escalation after myocardial infarction in the TALOS-AMI trial

**DOI:** 10.1080/07853890.2026.2726716

**Published:** 2026-09-30

**Authors:** Seonghyeon Bu, Jaehyuk Jang, Sang Hyun Kim, Jaeho Byeon, Kwan Yong Lee, Gyu-Chul Oh, Sungmin Lim, Eun Ho Choo, Ik Jun Choi, Byung-Hee Hwang, Chan Joon Kim, Mahn-Won Park, Kiyuk Chang, for the TALOS-AMI Investigators

**Affiliations:** a Division of Cardiology, Department of Internal Medicine, Uijeongbu St. Mary’s Hospital, College of Medicine, The Catholic University of Korea, Seoul, Republic of Korea; b Catholic Research Institute for Intractable Cardiovascular Disease (CRID), College of Medicine, The Catholic University of Korea, Seoul, Republic of Korea; c Division of Cardiology, Department of Internal Medicine, Seoul St. Mary’s Hospital, College of Medicine, The Catholic University of Korea, Seoul, Republic of Korea; d Division of Cardiology, Department of Internal Medicine, Incheon St. Mary’s Hospital, College of Medicine, The Catholic University of Korea, Seoul, Republic of Korea; e Division of Cardiology, Department of Internal Medicine, Daejeon St. Mary’s Hospital, College of Medicine, The Catholic University of Korea, Seoul, Republic of Korea

**Keywords:** De-escalation, percutaneous coronary intervention, P2Y_12_ inhibitors

## Abstract

**Background:**

The clinical effects of dual antiplatelet therapy de-escalation after acute myocardial infarction may differ by age. We evaluated whether the efficacy and safety of de-escalation from ticagrelor to clopidogrel differed by age in stabilized patients after percutaneous coronary intervention (PCI).

**Patients and methods:**

This was a prespecified secondary analysis of the TALOS-AMI randomized trial. Patients event-free 1 month after PCI receiving aspirin–ticagrelor were randomly allocated to de-escalation (aspirin–clopidogrel) or continuation (aspirin–ticagrelor). The primary net clinical endpoint was a composite of major adverse cardiovascular events (cardiovascular death, myocardial infarction, stroke) and Bleeding Academic Research Consortium types 2, 3, or 5 bleeding at 1 year.

**Results:**

We included 2697 participants (mean age 60.0 ± 11.4 years; 16.8% women). Among patients aged <75 years (*n* = 2376), de-escalation reduced the primary net clinical endpoint (4.1% vs. 7.2%; adjusted hazard ratio (aHR), 0.54 [95% CI, 0.38–0.77]) and bleeding (2.8% vs. 4.9%; aHR, 0.54 [0.35–0.82]). Among patients aged ≥75 years, no significant differences were observed for the primary endpoint (6.4% vs. 11.6%; aHR0.54 [0.25–1.17]) or bleeding (3.2% vs. 7.9%; aHR, 0.41 [0.15–1.15]). Interaction testing showed no treatment effect modification by age for the primary endpoint (*p* for interaction = 0.978), MACE (*p* = 0.585), or BARC bleeding (*p* = 0.597). Among patients aged ≥75 years, 14/18 bleeding events occurred within 180 days.

**Conclusions:**

Among stabilized, event-free patients 1 month after PCI, no significant age–treatment interaction was observed; therefore, the efficacy and safety of de-escalation in older adults (≥75 years) remain uncertain.

**Trial registration:** ClinicalTrials.gov (NCT02018055).

## KEY MESSAGES

In this prespecified secondary analysis of the TALOS-AMI trial, clopidogrel-based de-escalation was associated with lower risks of the primary net clinical endpoint and BARC type 2, 3, or 5 bleeding among patients aged <75 years, while MACE did not differ significantly between treatment strategies. Among patients aged ≥75 years, no statistically significant between-group differences were observed for the primary net clinical endpoint, MACE, or BARC type 2, 3, or 5 bleeding. No statistically significant treatment-by-age interaction was detected using the prespecified 75 years cutoff, alternative 65 and 70 years cutoffs, or age as a continuous variable; the efficacy and safety of de-escalation specifically in older adults remain uncertain.

## Graphical Abstract

**Figure:**
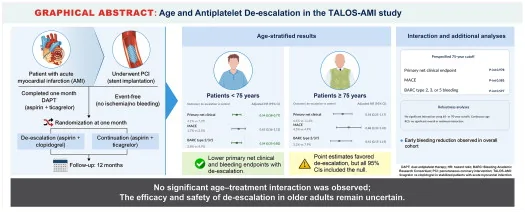

## Introduction

Dual antiplatelet therapy (DAPT) with aspirin and a potent P2Y_12_ inhibitor for at least 12 months is the standard of care after acute myocardial infarction (AMI), reducing the ischemic risk [1–4]. The ischemic effect of potent P2Y_12_ inhibition is most prominent in the early, high-risk period, whereas bleeding becomes the dominant concern during long-term maintenance therapy [5]. These time-dependent shifts in the ischemic and bleeding risks provide a rationale for de-escalation strategies that limit intensive DAPT to the initial phase and transition to less intensive therapy thereafter. This bleeding-avoidance rationale is reflected in contemporary acute coronary syndrome guidelines. The 2025 American College of Cardiology/American Heart Association guideline states that, 1 month after percutaneous coronary intervention (PCI), de-escalation from ticagrelor or prasugrel to clopidogrel and transition to single antiplatelet therapy for patients at a high bleeding risk may be reasonable (both are Class of Recommendation 2b and Level of Evidence B-R) [2], whereas the 2023 European Society of Cardiology guideline states that de-escalation to clopidogrel may be considered (Class 2b, Level A) and that aspirin or P2Y_12_ inhibitor monotherapy after 1 month may be considered for patients at a high bleeding risk (Class 2b, Level B) [1]. However, in older adults, bleeding and ischemic risks can increase concurrently, and whether the current standard therapy provides a comparable clinical benefit in this subset of the population remains unclear.

In the Ticagrelor vs. Clopidogrel in Stabilized Patients with Acute Myocardial Infarction (TALOS-AMI) study on patients stabilized after AMI and who underwent PCI, switching from aspirin–ticagrelor to aspirin–clopidogrel after 1 month resulted in superior clinical outcomes, compared with not switching, primarily as reductions in a composite of ischemic events (cardiovascular death, AMI, or stroke) or clinically relevant bleeding [6]. The improvement in outcomes was mainly driven by a reduction in bleeding events, without a significant increase in ischemic events. In this age-stratified secondary analysis of the TALOS-AMI study, patients were stratified according to a prespecified age cutoff of 75 years to assess whether the clinical effects of DAPT de-escalation differed by age. We hypothesized that de-escalation would confer a net clinical benefit across age groups by reducing bleeding without increasing ischemic events, although the magnitude and temporal pattern of benefit might differ in older patients.

## Patients and methods

### Study design

Details of the TALOS-AMI trial design, methodology, and main findings have been reported [6,7]. In brief, it was a multicenter, randomized, open-label, noninferiority study conducted at 32 hospitals in South Korea, enrolling patients with AMI who underwent PCI. All participants received aspirin plus ticagrelor for the first month after PCI. At 1 month, patients who were clinically stabilized without recurrent ischemia or bleeding were randomly assigned to either a de-escalation strategy with aspirin and clopidogrel or continuation of aspirin and ticagrelor. Patients were followed up for 12 months through scheduled outpatient visits and telephonic assessments. Data were obtained between February 14, 2014 and December 31, 2018, and follow-up concluded on January 21, 2021. The study protocol was registered at ClinicalTrials.gov (NCT02018055). The study protocol was approved by the institutional review boards of all participating centers, including the Institutional Review Board of Seoul St. Mary’s Hospital, The Catholic University of Korea (approval No. KC13MIMV0691), and the study was performed in accordance with the Declaration of Helsinki. All participants provided written informed consent.

In this age-stratified secondary analysis, we evaluated whether the clinical impact of DAPT de-escalation differs with age. The original trial prespecified 75 years as the threshold for defining the older age group, and age was modeled as a continuous variable in additional analyses. Spline curves were used to show the event rates and hazard ratios (HRs) for the de-escalation group versus the control group across the entire age range.

### Outcomes

Consistent with the original TALOS-AMI study, the primary net clinical endpoint of this analysis was a composite of major adverse cardiovascular events (MACEs) and Bleeding Academic Research Consortium (BARC) type 2, 3, or 5, at 12 months after the index PCI. A MACE was defined as a composite of cardiovascular death, myocardial infarction, and stroke. Key secondary outcomes were MACEs; a composite of BARC type 2, 3, or 5 bleeding; each individual type of BARC bleeding; and a composite of MACE and BARC type 3 or 5 bleeding. Other secondary outcomes comprised the individual components of the primary net clinical endpoint as well as all-cause mortality, ischemia-driven revascularization, and stent thrombosis.

### Statistical analysis

Continuous variables were reported as means ± standard deviations, whereas categorical variables were expressed as counts and percentages. Group comparisons for continuous variables were performed using the independent-samples t test or the Wilcoxon rank-sum test, as appropriate for the data distribution. Distributional assumptions were evaluated using visual inspection of histograms and quantile–quantile plots. Categorical variables were compared using the chi-square test. Time-to-event data were evaluated with Cox proportional-hazards models to assess the relationship between treatment strategy and clinical outcomes, including MACEs and bleeding events, with adjustment for age and sex. Interaction terms were tested within the Cox models to assess whether treatment effects varied according to age. We performed post hoc sensitivity analyses using alternative age cutoffs of 65 and 70 years. For each cutoff, treatment effects were estimated within the younger and older subgroups, and treatment-by-age-group interaction terms were tested in Cox proportional-hazards models. These alternative-cutoff analyses were considered exploratory. To further evaluate age as a continuous variable, treatment effects across the full age spectrum were examined using Cox regression models. To explore the temporal pattern of events, we summarized the cumulative distribution of event occurrence over time at prespecified time points. In addition, we performed a 180-day landmark analysis to evaluate whether the treatment effects differed between the early (≤180 days) and late (>180 days) periods. For the late-period analysis, only patients who remained event-free at the landmark time point were included, and follow-up was reinitiated from that time. Cox proportional-hazards models were applied separately within each period, and an interaction term was used to evaluate the differences in the treatment effects over time. As the original TALOS-AMI trial was powered for the overall randomized cohort rather than age-specific subgroup comparisons, a post hoc power analysis was performed for patients aged ≥75 years for the primary net clinical endpoint and BARC type 2, 3, or 5 bleeding by using a two-sided α of 0.05 and the observed subgroup event rates. Analyses were conducted using IBM SPSS Statistics (version 30; IBM Corp., Armonk, NY, USA) and R software (version 4.2.2; R Foundation for Statistical Computing, Vienna, Austria). A two-tailed significance threshold of 0.05 was applied for all statistical tests.

## Results

### Study population

The study sample had a mean (standard deviation) age of 60.0 (11.4) years; among them, 452 (16.8%) were female. Of the 2697 participants, 1349 were assigned to the aspirin plus clopidogrel (de-escalation) group and 1348 to the continued aspirin plus ticagrelor (active-control) group. All of them were included in this age-stratified analysis (Figure S1).

Most baseline demographic and procedural characteristics were balanced between the treatment groups in both the younger and older cohorts (Tables 1 and 2). However, the estimated glomerular filtration rate and infarct-related artery differed between the groups in the younger cohort, and the history of PCI differed between the groups in the older cohort.

**Table 1.** Baseline characteristics of patients according to age group.

	Age <75 y (*n* = 2376)			Age ≥75 y (*n* = 321)			
	De-escalation^a^ (*n* = 1192)	Active-control^b^ (*n* = 1184)	*p*	De-escalation (*n* = 157)	Active-control (*n* = 164)	*p*	*p* (diff)
Age, y	57.58 ± 9.45	57.31 ± 9.63	0.490	78.87 ± 3.73	78.49 ± 3.29	0.343	<0.001
Sex			0.228			0.829	<0.001
Male	1,041 (87.3%)	1,014 (85.6%)		91 (58.0%)	97 (59.1%)		
Female	151 (12.7%)	170 (14.4%)		66 (42.0%)	67 (40.9%)		
Body mass index, kg/m^2^	24.80 ± 3.04	24.76 ± 3.11	0.747	23.37 ± 3.14	22.90 ± 2.90	0.162	<0.001
CVD risk factor							
Hypertension	551 (46.2%)	553 (46.7%)	0.799	104 (66.2%)	110 (67.1%)	0.875	<0.001
Diabetes	306 (25.7%)	319 (26.9%)	0.474	56 (35.7%)	50 (30.5%)	0.324	0.011
Taking insulin	23 (1.9%)	24 (2.0%)	0.865	5 (3.2%)	4 (2.4%)	0.686	0.330
Dyslipidemia	497 (41.7%)	501 (42.3%)	0.746	66 (42.0%)	55 (33.5%)	0.116	0.140
Smoking			0.691			0.934	<0.001
Non-smoker	356 (29.9%)	339 (28.7%)		101 (64.3%)	103 (62.8%)		
Former smoker	192 (16.1%)	197 (16.7%)		30 (19.1%)	34 (20.7%)		
Current smoker	644 (54.0%)	647 (54.7%)		26 (16.6%)	27 (16.5%)		
Impaired kidney function^c^							
eGFR, mL/min/1.73 m^2^	87.76 ± 23.41	90.42 ± 25.08	0.008	75.31 ± 24.74	73.27 ± 25.28	0.469	<0.001
eGFR (<60 mL/min)	115 (9.9%)	94 (8.1%)	0.135	45 (29.2%)	51 (31.5%)	0.662	<0.001
History							
PCI	57 (4.8%)	47 (4.0%)	0.335	4 (2.5%)	13 (7.9%)	0.031	0.456
CABG	3 (0.3%)	0 (0.0%)	0.084	0 (0.0%)	1 (0.6%)	0.327	0.418
CVA	40 (3.4%)	43 (3.6%)	0.708	13 (8.3%)	7 (4.3%)	0.137	0.016
Clinical presentation			0.632			0.982	0.002
STEMI	662 (55.5%)	646 (54.6%)		72 (45.9%)	75 (45.7%)		
NSTEMI	530 (44.5%)	538 (45.4%)		85 (54.1%)	89 (54.3%)		
LVEF <40%	84 (7.2%)	77 (6.7%)	0.647	19 (12.3%)	16 (10.5%)	0.604	0.005

Data are presented as n (%) or means ± standard deviations. For smoking, data were missing for one patient in the younger active-control group. For eGFR (<60 m L/min), data were missing for 25 patients in the younger de-escalation group, 21 in the younger active-control group, 3 in the older de-escalation group, and 2 in the older active-control group. For LVEF <40%, data were missing for 21 patients in the younger de-escalation group, 33 in the younger active-control group, 3 in the older de-escalation group, and 11 in the older active-control group.

CABG: coronary artery bypass grafting; CVA: cerebrovascular accident; CVD: cardiovascular disease; eGFR: estimated glomerular filtration rate; LVEF: left ventricular ejection fraction; NSTEMI: non-ST-segment elevation myocardial infarction; PCI: percutaneous coronary intervention; STEMI: ST-segment elevation myocardial infarction.

^a^ The de-escalation group was administered aspirin plus clopidogrel.

^b^ The active-control group was administered aspirin plus ticagrelor.

^c^ Impaired kidney function was defined as an eGFR <60 mL/min/1.73 m^2^ of body-surface area at presentation.

**Table 2.** Procedural characteristics according to age.

	Age <75 y (*n* = 2376)			Age ≥75 y (*n* = 321)			
	De-escalation^a^ (*n* = 1192)	Active-control^b^ (*n* = 1184)	*p*	De-escalation (*n* = 157)	Active-control (*n* = 164)	*p*	*p* (diff)
Access site			0.452			0.968	0.692
Radial	584 (49.0%)	598 (50.6%)		82 (52.2%)	88 (53.7%)		
Femoral	594 (49.8%)	570 (48.2%)		73 (46.5%)	74 (45.1%)		
Both	14 (1.2%)	14 (1.2%)		2 (1.3%)	2 (1.2%)		
GP IIb–IIIa inhibitor	286 (24.0%)	290 (24.5%)	0.758	36 (22.9%)	32 (19.5%)	0.454	0.225
Infarct-related artery			0.018			0.881	0.089
LM	19 (1.6%)	20 (1.7%)		2 (1.3%)	4 (2.4%)		
LAD	610 (51.2%)	559 (47.3%)		75 (47.8%)	75 (45.7%)		
LCX	181 (15.2%)	242 (20.5%)		21 (13.4%)	22 (13.4%)		
RCA	381 (32.0%)	361 (30.5%)		59 (37.6%)	63 (38.4%)		
No. of treated vessels	1.33 ± 0.57	1.32 ± 0.55	0.901	1.34 ± 0.56	1.38 ± 0.60	0.600	0.267
Multivessel treatment			0.314			0.801	0.469
Two vessels	260 (21.8%)	280 (23.6%)		40 (25.5%)	42 (25.6%)		
Three vessels	64 (5.4%)	51 (4.3%)		7 (4.5%)	10 (6.1%)		
No. of stents	1.18 ± 0.42	1.17 ± 0.42	0.547	1.21 ± 0.42	1.18 ± 0.40	0.468	0.550
Total stent length, mm	29.68 ± 13.27	29.50 ± 13.74	0.740	30.88 ± 12.55	30.10 ± 14.35	0.608	0.269
Stent diameter, mm	3.21 ± 0.45	3.19 ± 0.47	0.511	3.12 ± 0.42	3.14 ± 0.42	0.603	0.008
Optical coherence tomography	42 (3.5%)	31 (2.6%)	0.216	5 (3.2%)	4 (2.4%)	0.692	0.803
IVUS	298 (25.0%)	270 (22.8%)	0.236	35 (22.3%)	37 (22.6%)	0.931	0.554

Data are presented as n (%) or means ± standard deviations. For the access site, data were missing for two patients in the younger active-control group. For the infarct-related artery, data were missing for one patient in the younger de-escalation group and two in the younger active-control group.

GP: glycoprotein; IVUS: intravascular ultrasound; LAD: left anterior descending artery; LCX: left circumflex artery; LM: left main coronary artery; No.: number; OCT: optical coherence tomography; RCA: right coronary artery; No. of stents: number of stents for infarct-related artery.

^a^ The de-escalation group was administered aspirin plus clopidogrel.

^b^ The active-control group was administered aspirin plus ticagrelor.

### Outcomes in the prespecified age subgroups

Results for the primary and key secondary outcomes are shown in Table 3 and Figure 1, whereas those for other secondary outcomes are detailed in Table S1. Among patients aged <75 years, the incidence of the primary net clinical endpoint was lower in the de-escalation group than in the active-control group (49 [4.1%] vs. 85 [7.2%]; adjusted HR [aHR], 0.54; 95% confidence interval [CI], 0.38–0.77; *p* < 0.001). The incidence of BARC type 2, 3, or 5 bleeding, a key secondary outcome, was also lower in the de-escalation group versus the active-control group (33 [2.8%] vs. 58 [4.9%] events; aHR, 0.54; 95% CI, 0.35–0.82; *p* = 0.004). In contrast, the incidence of MACEs, a key secondary outcome, did not differ between strategies in the younger cohort (1.7% vs. 2.5%; aHR, 0.63; 95% CI, 0.36–1.12; *p* = 0.11). For other secondary outcomes among patients aged <75 years, the de-escalation and active-control groups did not differ in terms of all-cause death (7 [0.6%] vs. 6 [0.5%]; aHR, 1.10; 95% CI, 0.37–3.25; *p* = 0.876), any myocardial infarction (10 [0.8%] vs. 17 [1.4%]; aHR, 0.56; 95% CI, 0.26–1.22; *p* = 0.145), target-vessel revascularization (16 [1.3%] vs. 15 [1.3%]; aHR, 1.00; 95% CI, 0.50–2.02; *p* = 0.998), or stent thrombosis (3 [0.3%] vs. 2 [0.2%]; aHR, 1.43; 95% CI, 0.24–8.55; *p* = 0.697).

**Figure 1.**
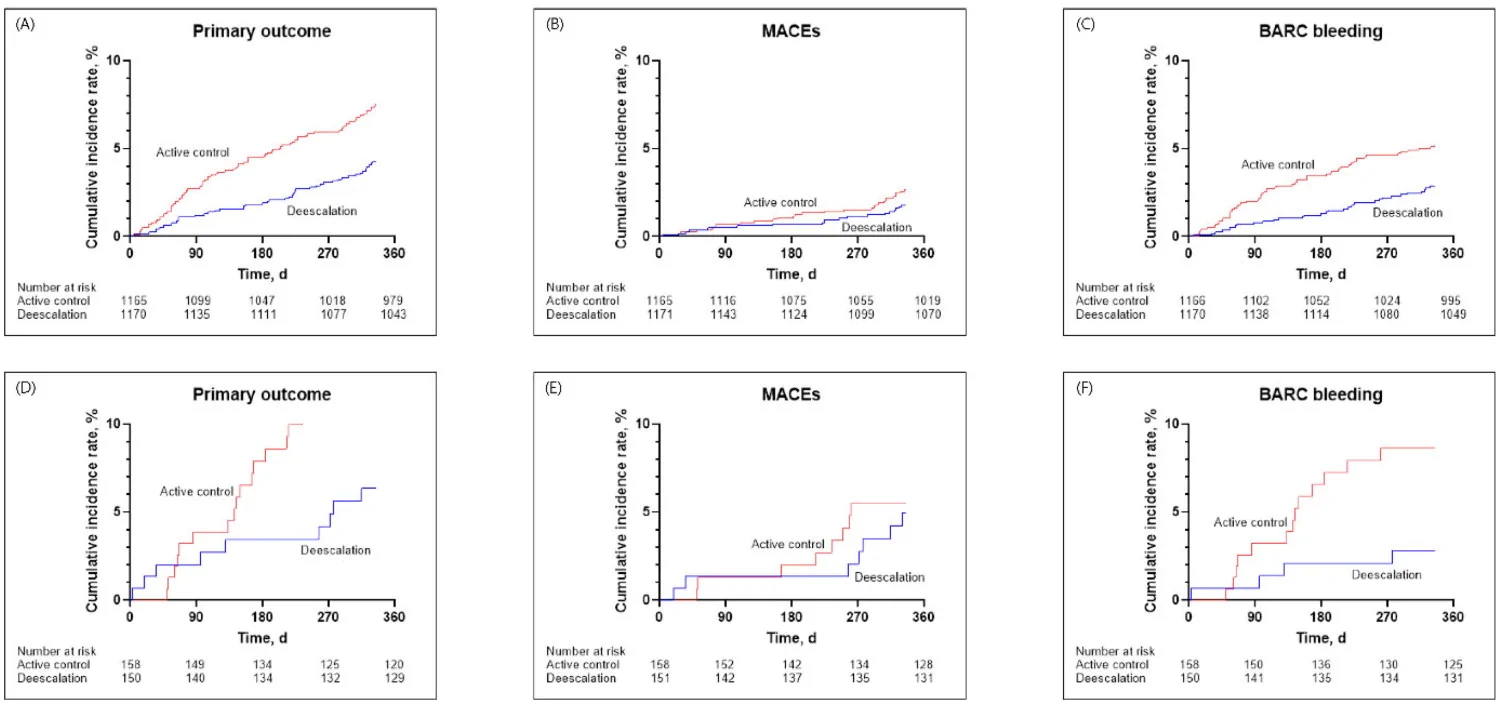
Kaplan–Meier curves stratified according to age. (A–C) represent patients aged <75 years, and (D–F) represent patients aged ≥75 years. The primary net clinical endpoint was a composite of cardiovascular death, myocardial infarction, stroke, and BARC type 2, 3, or 5 from 1 to 12 months after PCI. MACEs were defined as the composite of cardiovascular death, stroke, and myocardial infarction. BARC bleeding comprises type 2, 3, or 5 events according to the BARC criteria. BARC: Bleeding Academic Research Consortium; d, days; MACE: major adverse cardiovascular event; PCI: percutaneous coronary intervention.

**Table 3.** Primary and key secondary outcomes.

	Age <75 y (*n* = 2376)						Age ≥75 y (*n* = 321)							
	De-escalation (*n* = 1192)	Active-control (*n* = 1184)	Crude HR	*p*	Adjusted HR	*p*	De-escalation (*n* = 157)	Active-control (*n* = 164)	Crude HR	*p*	Adjusted HR	*p*	*p* int	*p* int (adj)
Primary net clinical endpoint	49 (4.1%)	85 (7.2%)	0.55 (0.39–0.79)	<0.001	0.54 (0.38–0.77)	<0.001	10 (6.4%)	19 (11.6%)	0.54 (0.25–1.16)	0.115	0.54 (0.25–1.17)	0.120	0.942	0.978
MACE	20 (1.7%)	30 (2.5%)	0.64 (0.37–1.14)	0.128	0.63 (0.36–1.12)	0.115	7 (4.5%)	8 (4.9%)	0.89 (0.32–2.45)	0.821	0.88 (0.32–2.43)	0.801	0.584	0.585
BARC bleeding type														
2, 3, 5	33 (2.8%)	58 (4.9%)	0.55 (0.36–0.84)	0.006	0.54 (0.35–0.82)	0.004	5 (3.2%)	13 (7.9%)	0.40 (0.14–1.13)	0.085	0.41 (0.15–1.15)	0.089	0.573	0.597
3, 5	12 (1.0%)	20 (1.7%)	0.58 (0.28–1.19)	0.135	0.56 (0.28–1.16)	0.117	3 (1.9%)	8 (4.9%)	0.39 (0.10–1.47)	0.166	0.39 (0.10–1.48)	0.168	0.600	0.603
2	23 (1.9%)	43 (3.6%)	0.52 (0.31–0.86)	0.011	0.50 (0.30–0.84)	0.008	4 (2.5%)	7 (4.3%)	0.62 (0.18–2.10)	0.431	0.64 (0.19–2.17)	0.470	0.820	0.790
3	12 (1.0%)	20 (1.7%)	0.58 (0.28–1.19)	0.135	0.56 (0.28–1.16)	0.117	3 (1.9%)	8 (4.9%)	0.39 (0.10–1.47)	0.166	0.39 (0.10–1.48)	0.168	0.600	0.603
5	0 (0.0%)	0 (0.0%)					1 (0.6%)	0 (0.0%)					>0.99	>0.99
MACE and BARC bleeding type 3/5	28 (2.3%)	47 (4.0%)	0.57 (0.36–0.92)	0.020	0.56 (0.35–0.90)	0.016	8 (5.1%)	14 (8.5%)	0.59 (0.25–1.40)	0.226	0.59 (0.25–1.40)	0.228	0.981	0.970

Adjustments were for age and sex.

adj: adjusted; BARC: Bleeding Academic Research Consortium; HR: hazard ratio; int: interaction; MACE: major adverse cardiovascular event.

Among patients aged ≥75 years, no statistically significant between-group differences were observed for the primary net clinical endpoint (10 [6.4%] vs. 19 [11.6%]; aHR, 0.54; 95% CI, 0.25–1.17; *p* = 0.120), MACEs (7 [4.5%] vs. 8 [4.9%]; aHR, 0.88; 95% CI, 0.32–2.43; *p* = 0.801), or BARC type 2, 3, or 5 bleeding (5 [3.2%] vs. 13 [7.9%]; aHR, 0.41; 95% CI, 0.15–1.15; *p* = 0.089). Formal interaction testing showed no evidence that the treatment effect differed between patients aged <75 and ≥75 years for the primary net clinical endpoint (*p* for interaction = 0.978), MACE (*p* for interaction = 0.585), or BARC type 2, 3, or 5 bleeding (*p* for interaction = 0.597). For other secondary outcomes among patients aged ≥75 years, no differences were observed between the groups in terms of all-cause mortality (4 [2.5%] vs. 4 [2.4%]; aHR, 1.01; 95% CI, 0.25–4.05; *p* = 0.991), any myocardial infarction (2 [1.3%] vs. 3 [1.8%]; aHR, 0.65; 95% CI, 0.11–3.94; *p* = 0.642), and target-vessel revascularization (1 [0.6%] vs. 2 [1.2%]; aHR, 0.51; 95% CI, 0.05–5.70; *p* = 0.588).

### Temporal distribution of events and 180-day landmark analysis

For BARC type 2, 3, or 5 bleeding, 14/18 events (77.8%) in patients aged ≥75 years occurred within 180 days of group allocation, compared with 54/91 events (59.3%) among patients aged <75 years (Table S2). In the 180-day landmark analysis (Table S3, Figures 2 and 3), de-escalation was associated with a lower risk of BARC bleeding during the first 180 days in the overall cohort (aHR, 0.40; 95% CI, 0.24–0.67) and in patients aged <75 years (aHR, 0.39; 95% CI, 0.22–0.70). No heterogeneity was observed between the time periods (*p* for interaction >0.05). Among patients aged ≥75 years, no differences were observed between the de-escalation and active-control groups in either period (≤180 days: aHR, 0.43; 95% CI, 0.14–1.39; >180 days: aHR, 0.32; 95% CI, 0.03–3.09; *p* for interaction = 0.840).

**Figure 2.**
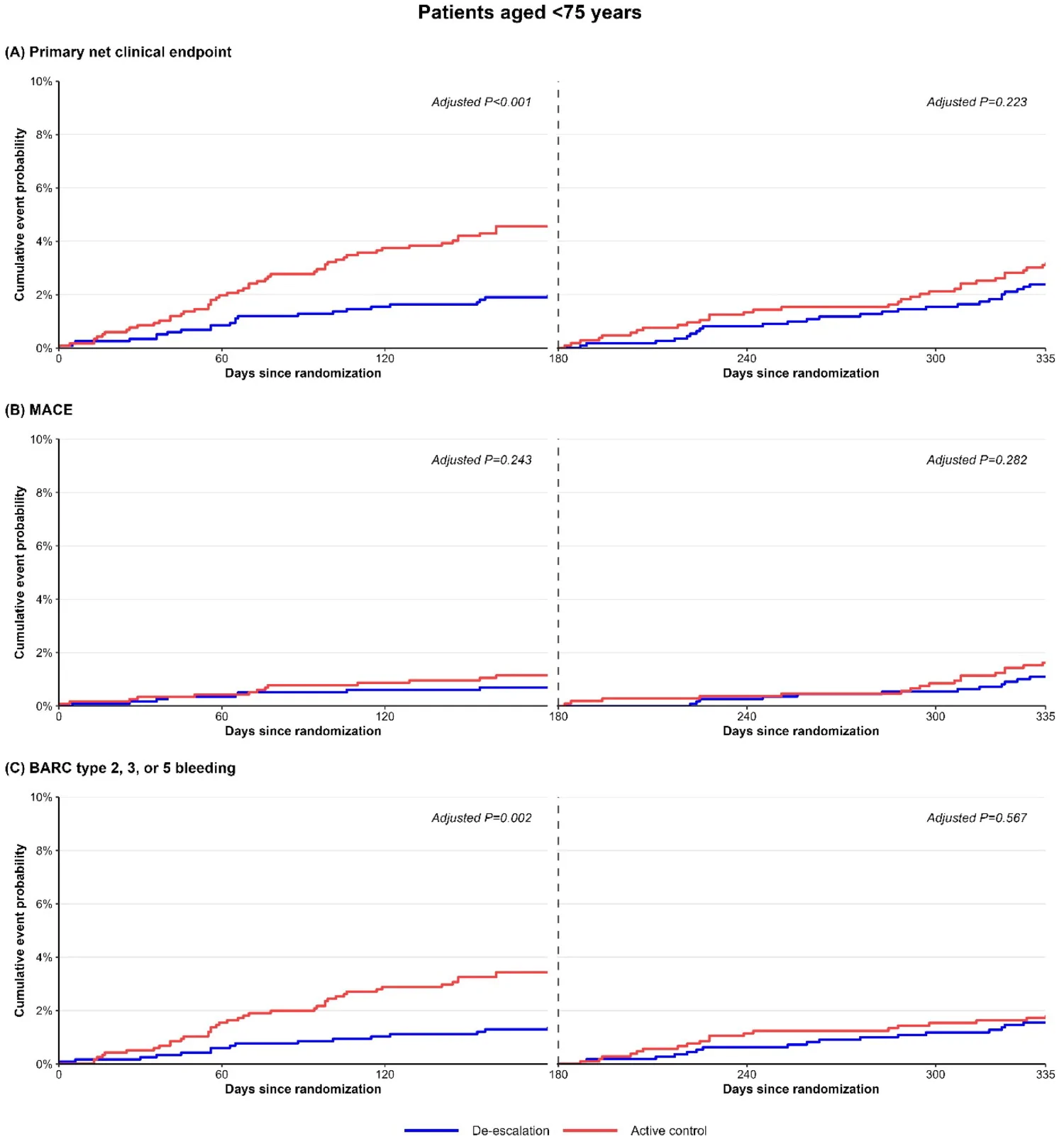
Kaplan–Meier curves for the 180-day landmark analysis in patients aged <75 years. (A) Primary net clinical endpoint, (B) MACE, and (C) BARC type 2, 3, or 5 bleeding. For each outcome, the left-hand plot shows the cumulative event incidence from randomization through day 180. The right-hand plot shows the conditional cumulative event incidence after day 180 among participants who were event-free and remained under observation at the landmark. Event probability was reset to zero at day 180, whereas the x-axis retains the time elapsed since randomization. The dashed vertical line marks the 180-day landmark. Displayed *p* values were derived from Cox proportional hazards models adjusted for age and sex. The primary net clinical endpoint was a composite of cardiovascular death, myocardial infarction, stroke, and BARC type 2, 3, or 5 bleeding. MACE was defined as a composite of cardiovascular death, myocardial infarction, and stroke. BARC bleeding comprised type 2, 3, or 5 bleeding events according to the BARC criteria. BARC: Bleeding Academic Research Consortium; MACE: major adverse cardiovascular event; PC: percutaneous coronary intervention.

**Figure 3.**
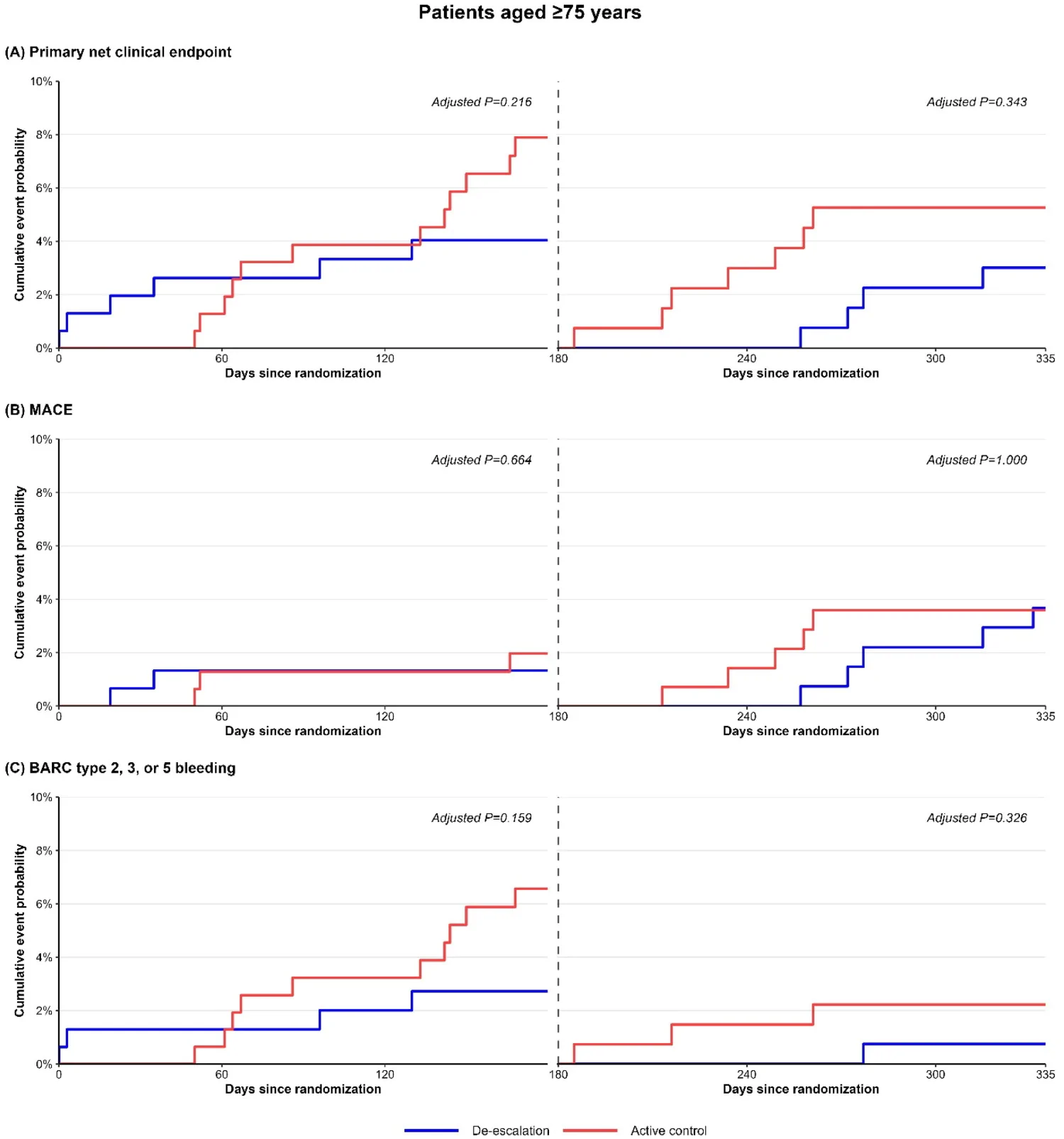
Kaplan–Meier curves for the 180-day landmark analysis in patients aged ≥75 years. (A) Primary net clinical endpoint, (B) MACE, and (C) BARC type 2, 3, or 5 bleeding. For each outcome, the left-hand plot shows the cumulative event incidence from randomization through day 180. The right-hand plot shows the conditional cumulative event incidence after day 180 among participants who were event-free and remained under observation at the landmark. Event probability was reset to zero at day 180, whereas the x-axis retains the time elapsed since randomization. The dashed vertical line marks the 180-day landmark. Displayed *p* values were derived from Cox proportional hazards models adjusted for age and sex. The primary net clinical endpoint was a composite of cardiovascular death, myocardial infarction, stroke, and BARC type 2, 3, or 5 bleeding. MACE was defined as a composite of cardiovascular death, myocardial infarction, and stroke. BARC bleeding comprised type 2, 3, or 5 bleeding events according to the BARC criteria. BARC: Bleeding Academic Research Consortium; MACE: major adverse cardiovascular event; PCI: percutaneous coronary intervention.

#### Sensitivity analyses using alternative age cutoffs

In post hoc sensitivity analyses, alternative definitions of older age did not change the findings. Among patients aged ≥65 years, the primary net clinical endpoint occurred in 30 of 473 patients (6.3%) in the de-escalation group and 44 of 475 patients (9.3%) in the active-control group (adjusted HR, 0.66; 95% CI, 0.41–1.04; *p* = 0.074). Among patients aged ≥70 years, the corresponding event rates were 7.2% and 9.8%, respectively (adjusted HR, 0.72; 95% CI, 0.41–1.24; *p* = 0.235). No statistically significant treatment-by-age interaction was detected using either the 65-year cutoff (*p* for interaction = 0.275) or the 70-year cutoff (*p* for interaction = 0.239). Similarly, no significant interactions were observed for MACE or BARC type 2, 3, or 5 bleeding using either cutoff (all adjusted *p* for interaction ≥ 0.094; Table S4).

## Analysis of age-dependent effect with age as continuous variable

When age was modeled as a continuous variable, the predicted cumulative event rates generally increased with advancing age. However, no statistically significant treatment-by-age interaction was observed for the primary net clinical endpoint, MACE, or BARC type 2, 3, or 5 bleeding (*p* for overall RCS interaction = 0.571, 0.945, and 0.329, respectively). No significant nonlinear interaction was identified (*p* for nonlinearity = 0.329, 0.877, and 0.199, respectively; Figure S2).

## Discussion

In this prespecified age-stratified analysis of the TALOS-AMI trial, no statistically significant treatment-by-age interaction was identified using the prespecified 75-year cutoff, alternative age cutoffs, or age as a continuous variable. Among patients aged <75 years, de-escalation was associated with lower risks of the primary net clinical endpoint and BARC type 2, 3, or 5 bleeding, whereas MACE did not differ significantly between treatment strategies. Among patients aged ≥75 years, no statistically significant between-group differences were observed for the primary net clinical endpoint, MACE, or BARC type 2, 3, or 5 bleeding. Similarly, the analyses using age cutoffs of 65 and 70 years did not establish a treatment benefit within the respective older subgroups. Although no statistically significant treatment-by-age interaction was detected, this should not be interpreted as proof of equivalent treatment effects across age groups. Given the limited number of events among older participants, the efficacy and safety of de-escalation specifically in older adults remain uncertain.

With the aging of the global population, older adults have become a major subject of interest in cardiovascular research. Older adults are recognized as a high-bleeding-risk population, with age being an independent predictor of bleeding, regardless of other comorbidities [8–14]. In the POPular AGE trial involving patients aged ≥70 years with non-ST-elevation acute coronary syndrome, use of clopidogrel from the time of PCI was associated with lower bleeding rates, compared with ticagrelor, without a corresponding increase in ischemic events [15]. These findings support clopidogrel de-escalation as a favorable strategy for older patients with an elevated bleeding risk.

Notably, even among stabilized patients allocated to a treatment group 1 month after PCI, bleeding events among those aged ≥75 years were concentrated early during follow-up: approximately three-quarters of bleeding events in that group occurred within the first 180 days of randomization. Consistent with this temporal distribution, the 180-day landmark analysis in the overall cohort showed that the bleeding benefit of de-escalation was most apparent within the first 180 days of randomization, whereas no significant between-group difference was observed thereafter, although no significant heterogeneity occurred in treatment effects between the time periods. This pattern supports the hypothesis that bleeding-avoidance strategies may warrant consideration earlier in the post-PCI course.

Several factors may explain the early bleeding hazard observed in this study. First, the early post-PCI phase is characterized by the use of potent DAPT and additional anticoagulants or glycoprotein IIb/IIIa inhibition. This should be viewed in light of the comorbidity burden of older adults, including kidney impairment, a low body mass index [16], frailty, baseline anemia, vascular fragility, and polypharmacy. Second, bleeding related to vascular access may occur more frequently in older adults, potentially reflecting age-related changes, such as endothelial dysfunction, increased arterial fragility, and progressive vascular degeneration [17–19]. Age alone is an incomplete measure of bleeding risk and should not be used as the sole criterion for selecting an antiplatelet strategy. Although absolute event rates generally increased with age, the continuous-age analyses did not demonstrate statistically significant treatment-effect modification. Age should therefore be interpreted together with frailty, renal function, anemia, body weight, prior bleeding, and other clinical measures of bleeding risk.

This study has some limitations. First, as a prespecified secondary analysis of data derived from the TALOS-AMI trial, the sample of participants aged ≥75 years was relatively small (*n* = 321, 11.9% of the overall cohort). Using a two-sided α of 0.05 and the observed treatment effects in this subgroup (primary net clinical endpoint: 6.4% vs. 11.6%; aHR, 0.54; BARC type 2, 3, or 5 bleeding: 3.2% vs. 7.9%; aHR, 0.41), the post hoc power was approximately 38% for the primary net clinical endpoint and 47% for bleeding. Under these assumptions, approximately 83 primary-endpoint events and 40 bleeding events would have been required to achieve 80% power, whereas only 29 and 18 events, respectively, were observed. Therefore, the absence of statistical significance in the ≥75-year subgroup should be interpreted as reflecting limited precision, rather than as an evidence of no treatment effect. Second, the landmark and temporal distribution analyses were exploratory in nature. The post-landmark analysis was conditional on patients being event-free and monitored at day 180, which might have introduced ­selection bias.

A dedicated randomized controlled trial focused on patients aged ≥75 years is warranted to define the optimal timing of de-escalation after PCI and to provide adequate power for both bleeding and ischemic outcomes. Future studies should also be conducted to determine whether precision-based approaches, such as frailty assessment and platelet function testing, can be used to personalize the de-escalation timing in older adults, because the generalizability of the present findings is limited to stabilized patients who have been event-free for 1 month after PCI.

## Conclusions

In this prespecified secondary analysis of the TALOS-AMI trial, no statistically significant treatment-by-age interaction was detected across prespecified, alternative-cutoff, or continuous-age analyses. Therefore, the efficacy and safety of de-escalation specifically in older adults remain uncertain. Notably, 77.8% of bleeding events in older adults occurred within the first 180 days, supporting the future prospective evaluation of earlier bleeding-avoidance strategies in this high-risk population.

## Supplementary Material

Supplementary Material_revision_1.docx

## Data Availability

Individual participant data will not be available until completion of follow-up in the TALOS-AMI trial. Thereafter, deidentified data may be shared with qualified investigators upon reasonable request to the corresponding author, in accordance with institutional policy and applicable ethical requirements.
