# Supplementary material for: Age and antiplatelet de-escalation after myocardial infarction in the TALOS-AMI trial
Source: Ann Med. 2026 Sep 30;58(1):2726716. doi: 10.1080/07853890.2026.2726716 (PMC13629817; doi:10.1080/07853890.2026.2726716)
Supplement: Supplementary file 1 [file IANN_A_2726716_SM3655.docx]

**Supplemental Material**

**List**

**Table S1. Other secondary outcomes**

**Table S2. Cumulative distribution of events over time**

**Table S3. Landmark analysis**

**Table S4. Primary and key secondary outcomes according to alternative age cutoffs**

**Figure S1. CONSORT flow diagram**

**Figure S2. Spline curves of the event rates and hazard ratios across the continuous age spectrum**

**Table S1. Other secondary outcomes**

|  | Age <75 y (n = 2376) | | | | | | Age ≥75 y (n = 321) | | | | | |  |  |
| --- | --- | --- | --- | --- | --- | --- | --- | --- | --- | --- | --- | --- | --- | --- |
|  | De-escalation (n=1,192) | Active-control (n=1,184) | Crude HR | P | Adjusted HR | P | De-escalation (n=157) | Active-control (n=164) | Crude HR | P | Adjusted HR | P | P int | P int (adj) |
| Death |  |  |  |  |  |  |  |  |  |  |  |  |  |  |
| All-cause | 7 (0.6%) | 6 (0.5%) | 1.13 (0.38–3.36) | 0.829 | 1.10 (0.37–3.25) | 0.876 | 4 (2.5%) | 4 (2.4%) | 1.04 (0.26–4.16) | 0.956 | 1.01 (0.25–4.05) | 0.991 | 0.931 | 0.948 |
| Cardiovascular | 4 (0.3%) | 4 (0.3%) | 0.97 (0.24–3.88) | 0.966 | 0.96 (0.24–3.83) | 0.951 | 2 (1.3%) | 2 (1.2%) | 1.04 (0.15–7.41) | 0.966 | 0.97 (0.14–6.92) | 0.972 | 0.953 | 0.930 |
| MI |  |  |  |  |  |  |  |  |  |  |  |  |  |  |
| Any MI | 10 (0.8%) | 17 (1.4%) | 0.57 (0.26–1.24) | 0.154 | 0.56 (0.26–1.22) | 0.145 | 2 (1.3%) | 3 (1.8%) | 0.69 (0.11–4.10) | 0.678 | 0.65 (0.11–3.94) | 0.642 | 0.853 | 0.857 |
| Spontaneous | 7 (0.6%) | 11 (0.9%) | 0.61 (0.24–1.58) | 0.311 | 0.62 (0.24–1.59) | 0.315 | 2 (1.3%) | 3 (1.8%) | 0.69 (0.11–4.10) | 0.678 | 0.65 (0.11–3.94) | 0.642 | 0.916 | 0.932 |
| Periprocedural | 3 (0.3%) | 6 (0.5%) | 0.48 (0.12–1.93) | 0.303 | 0.46 (0.11–1.83) | 0.267 | 0 (0.0%) | 0 (0.0%) |  |  |  |  | 0.999 | 0.999 |
| Target-vessel MI | 7 (0.6%) | 7 (0.6%) | 0.96 (0.34–2.74) | 0.943 | 0.94 (0.33–2.69) | 0.910 | 0 (0.0%) | 1 (0.6%) | 0.02 (0–152035) | 0.613 | 0 (0–NA) | 0.978 | 0.978 | 0.985 |
| Stroke | 6 (0.5%) | 9 (0.8%) | 0.65 (0.23–1.83) | 0.414 | 0.64 (0.23–1.80) | 0.392 | 3 (1.9%) | 4 (2.4%) | 0.77 (0.17–3.43) | 0.728 | 0.76 (0.17–3.39) | 0.715 | 0.849 | 0.876 |
| Revascularization |  |  |  |  |  |  |  |  |  |  |  |  |  |  |
| TVR | 16 (1.3%) | 15 (1.3%) | 1.03 (0.51–2.08) | 0.942 | 1.00 (0.50–2.02) | 0.998 | 1 (0.6%) | 2 (1.2%) | 0.51 (0.05–5.60) | 0.580 | 0.51 (0.05–5.70) | 0.588 | 0.589 | 0.614 |
| TLR | 13 (1.1%) | 8 (0.7%) | 1.57 (0.65–3.78) | 0.318 | 1.51 (0.63–3.64) | 0.360 | 1 (0.6%) | 1 (0.6%) | 1.02 (0.06–16.34) | 0.988 | 1.07 (0.07–17.23) | 0.963 | 0.777 | 0.807 |
| NTVR | 15 (1.3%) | 18 (1.5%) | 0.80 (0.40–1.59) | 0.529 | 0.80 (0.40–1.58) | 0.512 | 3 (1.9%) | 4 (2.4%) | 0.77 (0.17–3.43) | 0.728 | 0.77 (0.17–3.47) | 0.738 | 0.955 | 0.966 |
| Stent thrombosis | 3 (0.3%) | 2 (0.2%) | 1.44 (0.24–8.64) | 0.688 | 1.43 (0.24–8.55) | 0.697 | 0 (0.0%) | 1 (0.6%) | 0.02 (0–152035) | 0.613 | 0 (0–NA) | 0.978 | 0.986 | 0.991 |

adj., adjusted; HR, hazard ratio; int., interaction; MI, myocardial infarction; NA, nor available; NTVR, non-target vessel revascularization; TLR, target lesion revascularization; TVR, target vessel revascularization

**Table S2. Cumulative distribution of events over time**

| Primary outcome | 30 d | 60 d | 90 d | 180 d | 335 d | Mean, d |
| --- | --- | --- | --- | --- | --- | --- |
| Age <75 y (n=2,376) | 14 (10.4%) | 33 (24.6%) | 47 (35.1%) | 74 (55.2%) | 134 (100%) | 159 |
| Age ≥75 y (n=321) | 3 (10.3%) | 6 (20.7%) | 10 (34.5%) | 18 (62.1%) | 29 (100%) | 148 |
| MACEs |  |  |  |  |  |  |
| Age <75 y (n=2,376) | 6 (12.0%) | 9 (18.0%) | 15 (30.0%) | 21 (42.0%) | 50 (100%) | 192 |
| Age ≥75 y (n=321) | 1 (6.7%) | 4 (26.7%) | 4 (26.7%) | 5 (33.3%) | 15 (100%) | 199 |
| BARC bleeding |  |  |  |  |  |  |
| Age <75 y (n=2,376) | 8 (8.8%) | 25 (27.5%) | 33 (36.3%) | 54 (59.3%) | 91 (100%) | 146 |
| Age ≥75 y (n=321) | 2 (11.1%) | 3 (16.7%) | 7 (38.9%) | 14 (77.8%) | 18 (100%) | 124 |

BARC, Bleeding Academic Research Consortium; MACE, major adverse cardiovascular event

**Table S3. Landmark analysis**

|  | Overall (n=2,697) | | | | | | | Age <75 y (n=2,376) | | | | | | | Age ≥75 y (n=321) | | | | | | |
| --- | --- | --- | --- | --- | --- | --- | --- | --- | --- | --- | --- | --- | --- | --- | --- | --- | --- | --- | --- | --- | --- |
|  | De-escalation (n=1,349) | Active-control (n=1,348) | Crude HR | P | Adjusted HR | P | P interaction (adj) | De-escalation (n=1,192) | Active-control (n=1,184) | Crude HR | P | Adjusted HR | P | P interaction (adj) | De-escalation (n=157) | Active-control (n=164) | Crude HR | P | Adjusted HR | P | P interaction (adj) |
| Primary end point |  |  |  |  |  |  | 0.163 |  |  |  |  |  |  | 0.134 |  |  |  |  |  |  | 0.968 |
| ≤180 d | 29 (2.1%) | 64 (4.7%) | 0.45 (0.29–0.69) | <0.001 | 0.44 (0.29–0.69) | <0.001 |  | 23 (1.9%) | 52 (4.4%) | 0.43 (0.26–0.70) | <0.001 | 0.42 (0.26–0.69) | <0.001 |  | 6 (3.8%) | 12 (7.3%) | 0.53 (0.20–1.42) | 0.207 | 0.54 (0.20–1.44) | 0.216 |  |
| >180 d | 30 (2.2%) | 40 (3.0%) | 0.71 (0.44–1.13) | 0.150 | 0.70 (0.44–1.13) | 0.141 |  | 26 (2.2%) | 33 (2.8%) | 0.74 (0.44–1.24) | 0.252 | 0.73 (0.43–1.22) | 0.224 |  | 4 (2.5%) | 7 (4.3%) | 0.55 (0.16–1.88) | 0.342 | 0.55 (0.16–1.89) | 0.343 |  |
| MACEs |  |  |  |  |  |  | 0.735 |  |  |  |  |  |  | 0.854 |  |  |  |  |  |  | 0.757 |
| ≤180 d | 10 (0.7%) | 16 (1.2%) | 0.62 (0.28–1.37) | 0.237 | 0.62 (0.28–1.36) | 0.232 |  | 8 (0.7%) | 13 (1.1%) | 0.61 (0.25–1.46) | 0.264 | 0.59 (0.25–1.43) | 0.243 |  | 2 (1.3%) | 3 (1.8%) | 0.70 (0.12–4.21) | 0.700 | 0.67 (0.11–4.05) | 0.664 |  |
| >180 d | 17 (1.3%) | 22 (1.6%) | 0.74 (0.39–1.40) | 0.354 | 0.74 (0.39–1.39) | 0.346 |  | 12 (1.0%) | 17 (1.4%) | 0.67 (0.32–1.41) | 0.295 | 0.67 (0.32–1.40) | 0.282 |  | 5 (3.2%) | 5 (3.0%) | 1.00 (0.29–3.45) | 0.998 | 1.00 (0.29–3.46) | 1.000 |  |
| BARC bleeding |  |  |  |  |  |  | 0.111 |  |  |  |  |  |  | 0.093 |  |  |  |  |  |  | 0.840 |
| ≤180 d | 20 (1.5%) | 49 (3.6%) | 0.40 (0.24–0.68) | <0.001 | 0.40 (0.24–0.67) | <0.001 |  | 16 (1.3%) | 39 (3.3%) | 0.40 (0.24–0.72) | 0.002 | 0.39 (0.22–0.70) | 0.002 |  | 4 (2.5%) | 10 (6.1%) | 0.43 (0.13–1.36) | 0.150 | 0.43 (0.14–1.39) | 0.159 |  |
| >180 d | 18 (1.3%) | 22 (1.6%) | 0.78 (0.42–1.45) | 0.425 | 0.77 (0.41–1.44) | 0.412 |  | 17 (1.4%) | 19 (1.6%) | 0.85 (0.44–1.63) | 0.614 | 0.83 (0.43–1.59) | 0.201 |  | 1 (0.6%) | 3 (1.8%) | 0.33 (0.03–3.17) | 0.337 | 0.32 (0.03–3.09) | 0.326 |  |

adj., adjusted; BARC, Bleeding Academic Research Consortium; HR, hazard ratio; MACE, major adverse cardiovascular event

**Table S4. Primary and key secondary outcomes according to alternative age cutoffs**

(A) 65 years cutoff

|  | age < 65 years (n = 1749) | | | | | | age >= 65 years (n=948) | | | | | |  |  |
| --- | --- | --- | --- | --- | --- | --- | --- | --- | --- | --- | --- | --- | --- | --- |
|  | De-escalation (n = 876) | Active control (n = 873) | Crude HR | p value | Adjusted HR | p value | De-escalation (n = 473) | Active control (n = 475) | Crude HR | p value | Adjusted HR | p value | p interaction | p interaction (adj) |
| Primary end point | 29 (3.3%) | 60 (6.9%) | 0.46 (0.30-0.72) | <0.001 | 0.46 (0.30-0.72) | <0.001 | 30 (6.3%) | 44 (9.3%) | 0.67 (0.42-1.06) | 0.090 | 0.66 (0.41-1.04) | 0.074 | 0.259 | 0.275 |
| MACE | 13 (1.5%) | 20 (2.3%) | 0.63 (0.31-1.26) | 0.191 | 0.62 (0.31-1.25) | 0.185 | 14 (3.0%) | 18 (3.8%) | 0.77 (0.38-1.54) | 0.452 | 0.76 (0.38-1.53) | 0.445 | 0.696 | 0.693 |
| BARC Bleeding |  |  |  |  |  |  |  |  |  |  |  |  |  |  |
| type 2, 3, 5 | 18 (2.1%) | 43 (4.9%) | 0.40 (0.23-0.70) | 0.001 | 0.40 (0.23-0.70) | 0.006 | 20 (4.2%) | 28 (5.9%) | 0.71 (0.40-1.25) | 0.232 | 0.69 (0.39-1.22) | 0.197 | 0.168 | 0.183 |

Adjusted HR; adjusted by age and sex.

(B) 70 years cutoff

|  | age < 70 years (n = 2088) | | | | | | age >= 70 years (n=609) | | | | | |  |  |
| --- | --- | --- | --- | --- | --- | --- | --- | --- | --- | --- | --- | --- | --- | --- |
|  | De-escalation (n = 1045) | Active control (n = 1043) | Crude HR | p value | Adjusted HR | p value | De-escalation (n = 304) | Active control (n = 305) | Crude HR | p value | Adjusted HR | p value | p interaction | p interaction (adj) |
| Primary end point | 37 (3.5%) | 74 (7.1%) | 0.48 (0.32-0.71) | <0.001 | 0.48 (0.32-0.70) | <0.001 | 22 (7.2%) | 30 (9.8%) | 0.73 (0.42-1.26) | 0.252 | 0.72 (0.41-1.24) | 0.235 | 0.229 | 0.239 |
| MACE | 17 (1.6%) | 25 (2.4%) | 0.66 (0.36-1.22) | 0.184 | 0.65 (0.35-1.21) | 0.173 | 10 (3.3%) | 13 (4.3%) | 0.76 (0.33-1.72) | 0.505 | 0.76 (0.33-1.73) | 0.506 | 0.791 | 0.796 |
| BARC Bleeding |  |  |  |  |  |  |  |  |  |  |  |  |  |  |
| type 2, 3, 5 | 22 (2.1%) | 52 (5.0%) | 0.41 (0.25-0.57) | <0.001 | 0.40 (0.25-0.67) | <0.001 | 16 (5.3%) | 19 (6.2%) | 0.84 (0.43-1.63) | 0.608 | 0.83 (0.43-1.61) | 0.577 | 0.088 | 0.094 |

Adjusted HR; adjusted by age and sex.

**Figure S1. CONSORT flow diagram**

**Figure S2. Spline curves of the event rates and hazard ratios across the continuous age spectrum.** (A)–(C) represent the event rates, and (D)–(F) represent the hazard ratios. The primary outcome was a composite of cardiovascular death, myocardial infarction, stroke, and BARC bleeding type 2, 3, or 5 from 1 to 12 months after percutaneous coronary intervention. A MACE was defined as a composite of cardiovascular death, stroke, and myocardial infarction. BARC bleeding comprised bleeding event type 2, 3, or 5 according to the BARC criteria. Dotted vertical line was 75 years. BARC, Bleeding Academic Research Consortium; MACE, major adverse cardiovascular event
